# Advances in Flavor Formation Mechanisms and Technological Regulation of Sun-Processed Tea

**DOI:** 10.3390/foods15193500

**Published:** 2026-09-30

**Authors:** Li Guo, Chenxin Xie, Feng Zhao, Zhi Lin, Haipeng Lyu

**Affiliations:** 1 Tea Research Institute, Chinese Academy of Agricultural Sciences, Hangzhou 310008, China; guoli@tricaas.com (L.G.); xiechenxin@tricaas.com (C.X.); zhaofeng@tricaas.com (F.Z.); lvhaipeng@tricaas.com (H.L.); 2 Genetics and Breeding of Special Economic Animals and Plants, Ministry of Agriculture and Rural Affairs, Hangzhou 310008, China

**Keywords:** sun-processed tea, sun withering, sun drying, flavor chemistry

## Abstract

The application of sun withering and sun drying in tea processing has been demonstrated to reduce energy consumption while endowing finished tea with unique flavor attributes. This study reviews recent progress in the sensory characterization, aroma chemistry, and the technological and environmental regulatory mechanisms underlying quality formation in sun-processed tea. Existing research has identified geraniol, linalool, decanal, and 1-octen-3-ol as the core aromatic components of sun-processed tea, among which 1-octen-3-ol contributes substantially to its characteristic sunlight-associated flavor profile. Furthermore, sun-induced photothermal coupling can regulate endogenous tea metabolism and mediate the differential conversion of flavor precursors across different tea categories. Precise control of light intensity, irradiation duration, ventilation and other environmental parameters, combined with tea-specific process regulation, is the core to developing characteristic flavors and stabilizing the quality of commercial sun-processed tea. Future research should focus on technological optimization, mechanistic investigation, and industrial translation of sun-processed tea technologies, so as to provide theoretical guidance for the innovative application of energy-saving and simplified sun exposure processing in the global tea industry.

## 1. Introduction

Light, a crucial environmental factor with dual functions of energy supply and signal regulation, has been widely applied and intensively investigated in tea production [1]. During tea processing, solar withering or solar drying not only facilitates the transformation and accumulation of endogenous metabolites in fresh tea leaves, enabling the development of unique flavor profiles, but also effectively reduces energy consumption and production costs, which aligns with the green, low-carbon, and environmentally sustainable processing philosophy. Many tea-growing regions in China still practice this traditional technology. For the convenience of research classification and academic discussion, tea products treated with sun irradiation during processing are collectively referred to as ‘sun-processed tea’ in this study. When sunlight is adopted during tea withering or drying, the corresponding process is termed ‘sun-withering’ or ‘sun-drying’. Sun-dried flavor is the characteristic flavor of tea developed by sunlight exposure during processing.

Sun irradiation accelerates moisture loss in tea leaves and induces biochemical transformations of endogenous compounds—particularly those governing color, taste, and aroma—thereby defining the distinctive quality profile of sun-processed tea. This sensory profile arises from the integrated effects of critical processing parameters, including light intensity and spectral composition, ambient airflow, and equipment design; each exhibit substantial site- and batch-specific variability. Under optimal light conditions, controlled solar exposure has been shown to significantly enhance flavor complexity and overall sensory quality [2,3,4,5,6,7,8]. Conversely, inadequate process control—such as excessive exposure duration, high leaf temperature, or unregulated irradiance—can trigger undesirable chemical changes, resulting in off-flavors including sun-staled odor, flatness, stuffiness, and sour or rancid notes, all of which compromise product quality [9,10,11,12,13,14,15]. On overcast or rainy days, when natural irradiance falls below the threshold required for consistent photochemical activation, targeted LED supplementation can effectively maintain the requisite photic environment and ensure process robustness [16].

Given the dual impacts of sun processing on tea quality and the current lack of clear understanding of its underlying regulatory mechanisms, this narrative review synthesizes recent global research advances, summarizes the flavor and quality characteristics of diverse categories of sun-processed tea, and analyzes the corresponding chemical basis and formation mechanisms. This review aims to establish a theoretical foundation for the precise quality regulation of sun-processed tea and provide guidance for future investigations in this field.

## 2. Flavor Quality Characteristics of Sun-Processed Tea

Flavor, a comprehensive perception integrating olfactory and gustatory signals [16], is a critical quality attribute that exerts a dominant influence on consumers’ purchasing decisions for tea. The formation and transformation of tea flavor compounds occur predominantly during tea processing, and the incorporation of solar treatment in key processing stages including withering, drying, and fermentation plays a pivotal role in shaping the characteristic flavor and overall quality of the final product [1,3,12].

### 2.1. Sun Withering

During the withering of fresh tea leaves, leaf tissues remain intact and metabolically active, allowing physiological processes including respiration and photosynthesis to persist. These activities are accompanied by complex metabolic transformations of intracellular components, which exert a decisive influence on the eventual formation of tea quality [17,18,19,20]. At present, the most widely adopted withering methods include indoor natural withering, heated withering, sun withering, and compound withering [21]. Sun withering refers to the process of spreading fresh tea leaves under natural outdoor sunlight to induce withering. This method is characterized by simple operation and short processing duration, but it requires strict control of sunlight intensity and ambient temperature [22]. Accordingly, many tea manufacturing enterprises employ a compound withering technique that integrates indoor hot-air withering and sun withering to mitigate the impact of weather fluctuations on processing stability [23]. As a key technological innovation in the processing of Chinese Congou black tea, sun withering historically significantly reduced the production cost of exported black tea and generated considerable economic benefits, and it is still retained in some oolong tea-producing regions of China to this day [24,25]. During the spreading and withering of tea leaves, moisture in tea buds and leaves gradually dissipates, while endogenous tea components undergo dynamic transformations that ultimately confer the finished tea with distinct flavor characteristics [26]. The effect of sun withering on tea quality is primarily reflected in two dimensions: aroma and taste. Previous studies have demonstrated that supplementary solar irradiation during the spreading process can effectively improve the aroma quality of green tea [16]. Compared with indoor natural withering, white tea processed via sun withering develops a richer, more prominent aroma profile [2,4,6]. Oolong tea acquires a typical floral and fruity aroma with long-lasting fragrance after sun withering treatment [5,7], while black tea correspondingly exhibits a rich, lingering aroma with distinct floral and fruity notes [3,8,14,27]. Collectively, existing evidence indicates that sun withering may promote the formation and accumulation of floral and fruity volatile compounds in fresh tea leaves.

Sun spreading or withering not only enhances the aroma quality of tea but also influences its taste characteristics. Green tea subjected to sun spreading improves freshness and briskness, along with lower astringency [28]. After sun withering, Baihao Yinzhen develops a mellower taste accompanied by slight bitterness, while Shoumei displays a purer flavor with noticeable sweetness [15]. The taste quality of oolong tea is improved after sun withering, characterized by a mellow, fresh, and brisk profile with a sweet aftertaste [7,29] and diminished bitterness and astringency [5]. Existing studies have revealed distinct differences in the effects of sun withering on the taste of black tea. Jiuqu Hongmei develops a stale off-flavor [14], while Keemun black tea shows increased bitterness and astringency with degraded taste quality. By contrast, Jinxuan black tea exhibits smooth mouthfeel, Fuyun 6 black tea possesses a strong and rich flavor [27], and Yunnan black tea presents a mellow and harmonious taste [8], with their quality improved to varying degrees. These findings indicate that sun withering generally contributes to the improvement of taste quality in white tea and oolong tea; however, its effects vary considerably among different types of black tea. For a comparative overview, the changes in aroma and taste induced by sun withering across different tea categories are summarized in Table 1.

### 2.2. Sun Drying

Drying is the final stage in primary tea processing [30]; in other words, by further reducing the moisture content of buds and leaves, it inhibits enzymatic activity and promotes significant changes in appearance. Moreover, under the influence of heat, complex physicochemical transformations occur, including moisture loss and enzyme inactivation, maillard reaction-derived heterocyclic aroma formation, catechin polymerization and epimerization, and lipid oxidation, collectively determining the color, flavor, and storage stability of the finished tea [10,11,31]. The main drying methods for tea include natural air drying, sun drying, hot-air drying, microwave drying, and vacuum freeze-drying [32]. Among these approaches, open sun drying, also referred to as natural solar drying, is a well-established traditional preservation/processing method. Sun-dried tea typically develops a distinctive “sun-dried flavor,” reminiscent of the aroma released by sun-dried straw [31]. The formation of this characteristic sun-dried flavor may be primarily attributed to ultraviolet radiation in sunlight converting oxygen in the air into ozone, which, in turn, oxidizes the endogenous components of tea [33]. The taste characteristics of sun-dried teas vary considerably across tea types and differ notably from those produced by other drying methods. For green tea, sun drying yields an intense bitter and astringent taste with a pronounced sun-dried flavor [34,35], whereas hot-air drying produces a fresher, less astringent cup with roasted or cereal notes, and vacuum freeze-drying retains the briskness closest to fresh leaves [31,35]. White tea dried under sunlight develops a unique sun-dried flavor with a fresh and brisk taste, although cultivar differences exist [36,37,38]; in contrast, hot-air drying yields a mellower and sweeter profile, while freeze-drying preserves a lighter, more delicate taste [36,37]. Among dark teas, sun-dried Pu’er often presents a pungent or herbal-medicine-like taste [10,39], whereas hot-air drying significantly reduces raw astringency and enhances smoothness [11]; Liubao tea retains a sun-dried aroma but offers a more mellow taste [40]. Black tea produced by sun drying displays a relatively plain taste with a pure aroma [41], while hot-air drying yields a stronger, fuller-bodied, and malty-sweet flavor [41]. Overall, compared with other methods, sun drying generally imparts a more astringent taste and a characteristic sun-dried flavor across most tea types. To further clarify the chemical basis underlying these taste differences, the key taste-active compounds and their characteristic tastes in sun-dried teas are summarized in Table 2.

### 2.3. Sun Fermentation

Existing literature reports on the application of solar irradiation technology in tea fermentation remain relatively limited. In exploring the processing technology of Jiuqu Hongmei, Guo et al. [14] adopted sun anaerobic fermentation and fermenter-based fermentation. Their findings indicated that Jiuqu Hongmei produced by sun fermentation developed a slightly stuffy taste and a faintly greenish aroma, with its taste, aroma, and liquor color being somewhat inferior to those of Hongmei tea fermented by machines. In summary, the application of sun irradiation technology to the tea withering process may be more effective than its use in drying or fermentation—particularly in enhancing the aroma profile of oolong and white teas.

## 3. Flavor Chemistry of Sun-Processed Tea

Sun-processed teas exhibit distinct flavor profiles, with fundamental chemical characteristics differing significantly across tea types. Although recent studies have advanced the understanding of flavor chemistry in sun-processed teas from diverse global regions, systematic analytical frameworks and comprehensive, evidence-based reviews of their flavor profiles remain notably scarce.

### 3.1. Aroma Chemistry Characteristics of Sun-Processed Tea

#### 3.1.1. Aroma Chemical Components of Sun-Processed Tea

Tea exposed to sunlight exhibits distinct aroma profiles compared with conventionally processed counterparts. As shown in Table 3, despite variation in tea type and geographical origin, sun-withered teas predominantly display floral and fruity aroma notes. Sun-dried teas may contribute to developing a characteristic sun-dried flavor, although some display floral and fruity notes.

To date, research on the aroma chemistry of sun-processed tea primarily focuses on quantifying volatile components and identifying key aroma-active compounds. As shown in Table 3, white tea manufactured via solar withering typically presents pronounced floral and fruity notes. This characteristic aroma is mainly attributed to the synergistic effect of geraniol, linalool, β-myrcene and other related compounds; the concentrations of these volatiles are generally significantly higher than those of white tea produced via natural withering [4,6]. In a study investigating the processing technology of Jiuqu Hongmei tea, Guo et al. [14] compared sun anaerobic fermentation and fermenter-based fermentation. Their results showed that Jiuqu Hongmei produced via sun fermentation developed a slightly stuffy taste and faint greenish notes, and its overall sensory quality encompassing taste, aroma, and liquor color was inferior to that of machine-fermented Hongmei tea.

Significant differences are observed in the aroma chemical characteristics between sun-dried tea and sun-withered tea. Compared with green tea processed by single hot-air drying, green tea prepared via the combined sun drying—hot air drying method not only contains a more diverse profile of alcohol compounds, but also exhibits significantly higher concentrations of terpenic alcohols, including linalool, trans-linalool oxide, 4-terpineol, 2-decen-1-ol, and nerolidol [34]. Similar results are reported by Ye et al. [35].

The aroma profile of sun-dried green tea is considerably complex, characterized by high concentrations of terpenes, alcohols, and ketones. This observation suggests that sun drying facilitates the accumulation of alcoholic aroma compounds in green tea. Huang et al. [37] observe a similar pattern in white tea processing: the relative concentrations of methyl salicylate, methyl benzoate, α-ionone, hexyl hexanoate, dihydroactinidiolide, benzyl alcohol, (E)-2-octenol, decanal, 1-octen-3-ol, 2,4-heptadienal, and 3,5-octadien-2-one are all higher in sun-dried white tea than in non-sun-dried white tea. Among these compounds, 1-octen-3-ol was characterized by mushroom-like, earthy, and fatty odor notes, and its contribution to the overall aroma profile was greater in fully sun-dried white tea than in samples processed by other methods. This compound is correlated with the characteristic sun-dried flavor profile of white tea. Kun et al. [31] reported that sun drying significantly increased the levels of 1-octen-3-ol, hexanal, and 1-pentanol in white tea. In particular, the concentration of 1-octen-3-one, which imparts a mushroom odor, increases ~20-fold.

Chen et al. [36] suggest that five compounds detected exclusively in sun-dried white tea—namely δ-cadinene, decane, octane, 3-methyldecane, and isoprene—are closely associated with the formation of sun-dried flavor. Furthermore, (Z)-2-hexenal, which has a relative odor activity value of ≥100, exhibits a significant positive correlation with sun-dried aroma. Zhao et al. [46] investigated the aroma characteristics of raw dark tea prepared with different drying methods. They observed that 48 flavor compounds, including nonanal, hexanal, heptadienal, and ethyl acetate, are present at significantly higher levels in sun-dried raw dark tea and act as its characteristic flavor substances.

Table 4 summarizes the key aroma-active compounds identified in the volatile fractions of sun-processed teas in recent studies, which are primarily classified into alcohols, aldehydes, ketones, esters and other chemical categories. Typical representatives of these compounds include linalool, geraniol, and β-ionone: linalool contributes sweet, woody, lily-like, and magnolia-like aromas; geraniol exhibits a pronounced rose-like aroma; and β-ionone presents violet and fruity notes [42]. Together, these compounds confer sun-processed teas with their characteristic floral and fruity sensory profiles.

#### 3.1.2. Variation in Aroma Components in Sun-Processed Tea

Sun withering duration significantly influences the aroma quality of oolong tea Tieguanyin. As shown in Table 3, Tieguanyin samples subjected to 0–60 min of sun withering exhibit prominent floral or fruity notes, while a burnt off-note emerges when the sun withering duration is extended to 120 min. You et al. [29] reported that the concentrations of α-farnesene, nerolidol, indole, and other volatile compounds in sun-withered Tieguanyin peak at 15 min and reach the lowest level at 120 min. The relative abundances of hexyl hexanoate (contributing green bean and fresh fruit aroma), jasmolactone (contributing milky and fruity aroma), hexyl butyrate (contributing sweet fruit aroma), and aromatic compounds including phenylacetaldehyde (contributing hyacinth-like sweet aroma), phenethyl alcohol (contributing rose-like floral aroma) and benzyl alcohol (contributing fresh aroma) remain high during the first 30 min of sun withering, but decrease gradually as withering progresses. Existing studies have demonstrated that short-duration sun withering promotes aroma formation in oolong tea. As withering time increases, the expression of genes related to aroma metabolism is downregulated, which is accompanied by a reduction in the content of corresponding aroma compounds [49]. Collectively, these findings indicate that an appropriate sun-withering duration is beneficial for improving the aroma quality of tea.

Sun-dried tea leaves generally develop a characteristic sunburnt off-odor [11,31,34,37,40], even though certain processing conditions or tea varieties can produce pronounced sweet and floral aromas [34,37]. However, the sun-burnt odor is relatively pungent and can overpower the desirable sweet and floral notes naturally present in tea leaves. The duration of sunlight exposure significantly influences the formation of sun-burnt odor. For instance, tea leaves exposed to 3 h of sunlight exhibit a more intense sun-burnt odor than those exposed for only 1 h [34]; furthermore, as sun-withering time extends, the original floral and fruity aromas of tea leaves gradually convert to sun-burnt odor [29]. For both sun-dried tea and sun-withered tea, prolonged sunlight exposure induces the formation of sun-burnt odor. There is a clear correlation between the duration of sunlight exposure and the intensity of sun-burnt odor in tea leaves: as exposure time increases, the sun-burnt odor gradually becomes more prominent and intensified; in contrast, short-term sun withering or drying treatments generally do not produce sun-burnt off-odor in tea leaves or only result in a very faint sun-burnt odor.

### 3.2. Chemical Characteristics of Taste in Sun-Processed Tea

#### 3.2.1. Chemical Components of Taste in Sun-Processed Tea

To date, no consistent conclusions have been reached regarding the taste quality of sun-withered tea. Even for the same tea type, the final products might exhibit markedly divergent sensory profiles—particularly in taste due to differences in tea plant cultivar, tenderness of fresh tea leaves, and other factors [14,15]. In comparison, sun-dried tea exhibits certain commonalities in taste quality, with a prominent astringent taste being the most notable characteristic [11,12,13,34]. The primary bitter-tasting compounds in tea include tea polyphenols, alkaloids, tea saponins, and bitter amino acids. Among these, polyphenolic compounds such as flavanones, flavanols, flavonols, and anthocyanins contribute to bitterness and astringency [42]. Ester-type catechins demonstrate the strongest empirical correlation with astringency perception and are consistently present at higher concentrations than their non-esterified counterparts within tea matrices [43]. Free amino acids primarily contribute to the umami taste [44], while soluble sugars, sweet amino acids, and sweet peptides serve as the main sweet-tasting compounds [42,50]. The taste of tea infusion represents a complex sensory perception, which is formed by the combination of various taste-active compounds and the interactions between different flavor-active substances.

Sun withering influences the taste quality of tea by altering the chemical composition of its constituents. In a comparative study investigating the impact of withering method on major taste-related metabolites in summer–autumn black tea, Zi et al. [8] reported that sun-withered samples exhibited significantly higher concentrations of tea polyphenols, whereas naturally withered samples showed markedly reduced levels of free amino acids and soluble sugars; caffeine content remained statistically invariant across both treatments. As a result, sun-withered black tea exhibits a mellower and smoother taste. A similar trend is observed in Keemun black tea: sun-withered samples show prominent sweetness and astringency, with significantly higher concentrations of catechins and soluble sugars than other tea samples [9]. Similar findings are reported for white tea and oolong tea. Sun-withered white tea has higher soluble sugar concentrations but significantly lower tea polyphenol concentrations than samples subjected to natural withering [15]. For Tieguanyin tea, a representative oolong tea, sun withering enhances the extraction yields of water-soluble solids and increases the abundance of key taste-active constituents, including free amino acids, soluble sugars, and flavonoids [7], thereby promoting the formation of fresh, brisk, and sweet flavors while reducing the concentrations of tea polyphenols, caffeine, and other compounds associated with bitterness and astringency. Ou et al. [51] report that sun-withered Dancong tea (an oolong tea) exhibits a mellow and brisk taste profile with a persistent sweet aftertaste; furthermore, it displays significantly elevated concentrations of free amino acids and caffeine relative to teas subjected to alternative withering methods, thereby underpinning the distinctive sensory quality characteristic of Dancong tea. Collectively, these studies indicate that the appropriate application of sun withering can improve tea taste quality. The underlying mechanism may be that sun withering reduces the concentrations of tea polyphenols and caffeine in tea leaves while increasing the concentrations of free amino acids and soluble sugars. These changes enhance the sweetness of the tea infusion and alleviate bitterness and astringency, thereby improving the overall taste quality of the tea [9].

Observable differences exist in the effects of the sun-drying method on the taste quality of different tea types. In white tea processing, a combined drying approach of sun drying and oven drying is preferred for tea drying, primarily because white tea treated with this combined method exhibits a fresher and brisker taste than those dried through pure oven drying or pure sun drying, along with higher concentrations of water extracts, tea polyphenols, and free amino acids [38]. By contrast, sun-dried dark tea exhibits a significantly more intense astringency and lower overall sensory quality scores relative to samples processed via hot-air drying, far-infrared drying, or other conventional thermal drying methods [11]. No significant difference is observed in water extract concentration between sun-dried dark tea and samples processed by other methods; however, sun-dried dark tea has lower concentrations of free amino acids and soluble sugars, higher concentrations of tea polyphenols and caffeine, and the highest total catechin concentration. No significant difference in astringency is observed between sun-dried yellow tea and conventionally dried samples. Nevertheless, sun-dried yellow tea exhibits slightly higher astringency and lower contents of tea polyphenols, free amino acids, soluble sugars, and caffeine [52]. Similarly, sun-dried black tea presents aggravated astringency with reduced tea polyphenol and caffeine concentrations [53].

#### 3.2.2. Variation in Taste Components of Sun-Processed Tea

Studies on the variation in taste components in sun-processed tea are still limited at that time. During sun drying of green tea, the concentration of water-soluble solids decreases significantly; catechins show an initial decrease followed by an increase; total flavonoids increase gradually; total free amino acids undergo an early reduction followed by a subsequent elevation, while the concentrations of theobromine and caffeine fluctuate irregularly [54]. In oolong tea subjected to solar radiation during withering, catechin content exhibits an initial decline followed by a partial recovery as withering duration extends [29]. Sun withering alleviates bitterness in black tea and produces a mellower infusion, primarily due to higher concentrations of water extracts and free amino acids together with lower tea polyphenol concentrations [27]. These findings suggest that sun treatment exerts divergent effects on the formation of taste components across different tea types.

## 4. Technological and Environmental Factors Influencing Flavor Formation in Sun-Processed Tea

### 4.1. Selective Use of Sunlight

The selective utilization of sunlight serves as a critical technical foundation for the production of sun-processed tea and is a core determinant of its flavor quality. Due to the presence of atmospheric water vapor, suspended particulates, cloud cover, and artificial shading, sunlight is scattered, diffused, or filtered before reaching tea leaves, which results in fluctuations in incident light intensity and ultimately leads to divergent effects on the final tea quality. Existing literature has confirmed that moderate light intensity facilitates the improvement of tea aroma quality, while excessively high intensity negatively impacts overall tea quality [55].

Green tea spread under yellow light exhibits superior aroma quality compared with that processed under red light and white light, with more prominent floral and chestnut-like notes [28]. For black tea processed with yellow-light withering [56], light intensity has only a minor effect on the appearance of dry tea and the quality of infused leaves, but exerts a significant impact on the color, aroma, and taste of the infusion. As light intensity increases, the redness of the infusion deepens, the taste becomes sweeter and mellower, and the sweet aroma becomes more pronounced. However, when light intensity exceeds 1000 lx, only marginal variations in black tea quality are detected. By contrast, results from a sun-withering experiment using fresh Dancong tea leaves [57] show that raw tea processed under 16,774 lx attains the best overall quality. Tea samples treated at 11,230 lx and 20,500 lx obtain comparable quality scores, while the sample exposed to 24,975 lx has the lowest score. Collectively, these findings indicate that higher light intensity during withering does not necessarily result in improved tea quality.

In addition to light intensity, exposure duration also influences tea quality to a certain extent. You et al. [29] report that floral and fruity aromas in sun-processed tea diminish when sun withering exceeds 60 min, which is consistent with the trend observed for natural withering [26]. Extended sun withering (120 min) further leads to the development of a sun-burnt odor in the final product. When light exposure exceeds 6 h, black tea develops a more pronounced fruity aroma and a cleaner, fresher taste, indicating a clear time-dependent effect of light exposure within a specific duration range [56]. During oolong tea processing, aroma accumulation does not show a linear positive correlation with sun withering duration; however, appropriate sun-withering facilitates the formation of the characteristic flavor profile of oolong tea [29,49]. Liupao tea produced with shorter sun-drying duration features a mellow taste and prominent floral aroma, while samples subjected to longer sun-drying exhibit a mellow base accompanied by undesirable greenish and sun-burnt aromas [40]. Taken together, these findings demonstrate that appropriate light exposure during the withering stage improves tea quality, while excessively long exposure may deteriorate tea quality and even induce undesirable odors.

The rational application of light irradiation across different tea processing stages leads to significant differences in the flavor quality of the final tea product. Tea samples with sun withering show a higher propensity to develop floral and fruity aroma characteristics, while tea processed via sun drying typically presents distinct sun-dried notes. These findings indicate that the appropriate implementation of sun treatments during tea processing facilitates the formation of desirable tea quality, whereas improper application may induce unfavorable quality outcomes. Sun-based processing technologies can be integrated with other processing methods and applied across multiple manufacturing stages of tea. In the fresh leaf withering stage, sun withering can be used independently or combined with other withering methods to form compound withering, which has been widely adopted in commercial tea production [8,16,58]. Existing studies have demonstrated that tea samples processed via compound withering and natural withering differ significantly in flavor profiles [59,60], confirming that sun withering exerts a substantial impact on the final quality of tea products. The mainstream sun-drying methods for tea include direct sun drying, combined oven-sun drying [35,38], and combined shade-sun drying [37]. Tea products obtained from these different drying patterns show subtle but distinguishable variations in flavor quality.

### 4.2. Environmental Factors

During tea processing, tea leaves are directly exposed to sunlight and are therefore inevitably affected by environmental factors, including temperature, relative humidity, and wind speed. Wind speed modulates the water loss rate of fresh tea leaves, which in turn alters the processing properties of withered leaves [61]. However, existing research focusing specifically on the effect of wind speed on sun withering remains scarce. Maintaining ambient temperature and humidity within appropriate ranges during processing contributes to the improvement of tea quality, while suboptimal conditions often lead to bitter and astringent taste and reduced aroma intensity [62,63,64]. Environmental humidity is a core regulatory factor for fresh leaf withering, as its level directly determines the rate of leaf water loss. Excessively low humidity triggers rapid dehydration of withered leaves, resulting in leaf reddening and overall quality deterioration [65]. To achieve desirable sun-processing performance, practical tea production generally requires appropriate seasonal selection based on the climatic characteristics of the production region [66]. Previous studies have demonstrated that maintaining a withering temperature between 25 °C and 35 °C promotes the formation of high-quality tea [66]. Favorable temperature and light conditions in spring contribute to the excellent overall quality of sun-withered tea. In contrast, the higher temperatures and significantly stronger light intensity in summer and autumn easily induce reddening of withered leaves, leading to increased bitterness and astringency as well as reduced freshness and briskness in the finished tea products [67,68,69].

## 5. Summary and Outlook

The distinct quality characteristics of sun-processed tea are closely shaped by its specific processing techniques. Current research primarily focuses on tea manufacturing processes and the regulatory mechanisms of flavor formation. Sun withering is a widely applied pre-treatment in the processing of black tea, white tea, oolong tea and other tea categories, and can effectively improve the sensory quality of finished tea products. In contrast, sun drying is more commonly used in the processing of green tea, white tea and dark tea, and is particularly suitable for fresh leaves harvested from large-leaf tea cultivars. While prolonged sunlight exposure reduces or even eliminates the floral and fruity aromas of tea, it enhances the characteristic sun-burnt note, which is associated with increased concentrations of the typical aroma compounds 1-octen-3-one and 1-octen-3-ol. Different tea types exhibit divergent changes in aroma composition after sun treatment, leading to inconsistent sensory outcomes of sun processing. As a result, sun-processed tea does not inherently present undesirable flavors. From the perspective of chemical composition, linalool, geraniol, β-ionone and other terpenoid compounds in sun-processed tea are the core aroma contributors to its floral and fruity notes, while elevated concentrations of bitter and astringent substances including ester catechins and caffeine result in an intensified bitter and astringent taste. In summary, sun withering can contribute to improving the flavor quality of white tea, black tea, and oolong tea. However, the water loss rate of semi-finished products must be strictly controlled during processing to prevent the “dead green” phenomenon caused by excessive rapid moisture reduction. Sun drying tends to induce off-odors in tea leaves, and this effect is more pronounced in high-moisture semi-finished products such as fermented leaves. Therefore, the standalone sun-drying process should be applied with caution for all tea types except sun-processed green tea.

In recent years, processing technologies for China’s six major tea categories have developed growing integration and mutual reference, giving rise to a diverse range of novel tea products. As a renewable and clean energy source, the application of solar radiation in tea processing significantly reduces energy consumption, generating both economic and ecological benefits, which is also consistent with the concept of low-carbon manufacture. Accordingly, sun-processed tea is characterized by a unique sensory flavor and high market value. Nevertheless, the production of high-quality sun-processed tea is constrained by suitable producing regions, standardized processing conditions, and matched supporting processing technologies.

Based on the current research progress of sun-processed tea, future research should focus on the following aspects. First, in terms of theoretical research, efforts should be made to deeply analyze the formation and transformation mechanisms of flavor compounds in sun-processed tea. Multiomics technologies should be applied to rapidly detect and identify key aromatic and taste-active compounds, so as to clarify the chemical basis underlying the formation of flavor quality. Further, the regulatory effects of different light qualities on the quality development of sun-processed tea should be systematically explored, and artificial light source equipment that can replace natural sunlight should be developed to break through the limitations imposed by natural climatic conditions. Second, in terms of technological development, systematic standardized research should be conducted on key processing parameters of sun-processed tea, including withering thickness, withering duration and drying time, as well as environmental factors such as temperature, humidity, wind speed and light intensity. On this basis, processing technologies should be optimized and integrated, and unified technical specifications for the industrial production of sun-processed tea should be established. In addition, extraction and preparation technologies for functional components from sun-processed tea should be developed to enable the development of high-value-added end products. Finally, in terms of industrial applications, the deep integration of sun-drying processes and traditional tea manufacturing procedures should be promoted, characteristic-flavored tea products should be developed, and standardized, large-scale production lines for sun-processed tea should be constructed.

## Figures and Tables

**Table 1:** The effects of solar withering on the aroma and taste profiles of different tea categories.

Tea Category	Withering Method	Changes in Aroma	Changes in Taste	References
Green tea	Sun withering/light-supplemented spreading	Significantly improved aroma quality, promoting the formation of floral and fruity volatiles	Enhanced freshness and briskness, reduced astringency	[16,28]
White tea	Sun withering	Richer and more pronounced aroma, with enhanced floral and fruity notes	Baihao Yinzhen: more mellow with slight bitterness; Shoumei: purer taste with obvious sweetness	[2,4,6,15]
Oolong tea	Sun withering (sun-drying)	Typical floral and fruity aromas are highlighted, with increased persistence	Overall taste quality improved: mellow, fresh, brisk with sweet aftertaste; bitterness and astringency significantly reduced	[5,7,29]
Black tea	Sun withering/combined withering	High and persistent aroma with obvious floral and fruity notes	Variety-specific effects: Enhancing type (Jinxuan, Yunyun No. 6, Yunnan black tea): smoother, richer, more mellow; Reducing type (Jiuqu Hongmei): stale off-flavor; Keemun: increased bitterness and astringency, decreased taste quality	[3,8,14,27]

**Table 2:** Key taste-active compounds and their characteristic tastes in sun-dried teas.

Tea Type	Key Taste-Active Compounds	Characteristic Taste	References
Green tea	Catechins (EGCG, ECG), flavonol glycosides, caffeine, amino acids (theanine)	Heavy bitter and astringent; pronounced sun-dried flavor; reduced freshness compared to freeze-dried tea	[31,34,35,42,43]
White tea	Catechins, theaflavins, thearubigins, soluble sugars, amino acids	Unique sun-dried flavor; fresh and brisk; cultivar-dependent sweetness/bitterness balance	[36,37,38,42]
Dark tea (Pu-er)	Theabrownins, gallic acid, low-residual catechins, soluble sugars	Pungent or herbal-medicine-like taste; thick and strong; reduced raw astringency	[10,39,42,44]
Dark tea (Liubao)	Catechins, theabrownins, soluble sugars, organic acids	Retains sun-dried aroma; relatively mellow and smooth	[40,42]
Black tea	Theaflavins, thearubigins, catechins (residual), amino acids, soluble sugars	Pure aroma; relatively plain taste; less full-bodied compared to hot-air dried tea	[41,42]

Note: EGCG, epigallocatechin gallate; ECG, epicatechin gallate.

**Table 3:** Summary of research results on aroma composition of sun-processed tea.

Key Technology	Name	Aroma Sensory Evaluation	Origin of Samples	Detection Method	Number of Compounds	References
Withering	Gongmei	Fresh, prominent pekoe	Ya’an, Sichuan	HS-SPME/GC-MS	68	[45]
	White tea	Intense floral, fruity	Dali, Yunnan	HS-SPME/GC-MS	131	[6]
	White tea	With floral notes	Nanping, Fujian	SAFE/GC-MS	175	[4]
	Tieguanyin	0 min: faint floral and fruity; 15 min: distinct, rich and persistent floral and fruity; 30 min: noticeable floral and fruity notes, relatively long-lasting; 60 min: slight fruity; 120 min: mild aroma with typical sun-burnt	Fujian	HS-SPME/GC-MS	99	[29]
	Black tea	Floral and fruity, with a honey-like aroma	Qimen, Anhui	SPME/GC-MS, SAFE/GC-MS	191	[3]
Drying	Green tea	with sun-burnt character	Chongqing	SPME/GC-MS	105	[34]
	Green tea	sun-burnt, hawthorn, sweet and floral	Yunnan	HS-SPME/GC-MS	84	[31]
	Green tea	slightly rough and pungent	Guangdong	SPME/GC-MS	27	[10]
	Green tea	Sweet, floral	Qimen, Anhui	HS-SPME/GC-MS	86	[35]
	White tea	Fresh and floral, with sun-burnt character	Dali, Yunnan	HS-SPME/GC-MS	133	[37]
	Dark tea	—	Anhua, Hunan	HS-GC-IMS	65	[46]
	Dark tea	Aged aroma, grassy	Kunming, Yunnan	HS-SPME/GC-MS	66	[39]
	Black tea	Floral, fruity, grassy, sweet, woody	Puer, Yunnan	HS-SPME/GC-MS	86	[47]

Note: HS-SPME, headspace solid-phase microextraction. GC-MS, gas chromatography-mass spectrometry. SAFE, solvent-assisted flavor evaporation. SPME, solid-phase microextraction.

**Table 4:** The key aroma components of sun-processed tea.

Name	Extraction Methods	Determining Methods	Key Volatile Component (OAV)	References
Gongmei (sun withering)	HS-SPME	GC-MS/OAV	β-ionone (1106.2), linalool (8.7), trans-2-decenal (1.3), nonanal (2.5), dimethyl sulfide (2.4),	[45]
White tea (sun withering)	SAFE	GC-MS/OAV	Dimethyl sulfide (210.9), (E,E)-2,4-heptadienal (122.8), geraniol (35.3), (E)-β-ionone (30.7), hexanal (25.9), (Z)-4-heptenal (14.5), linalool (11.1), β-myrcene (6.7), 2-Methyl-butanal (3.6), 1-Penten-3-one (3.0), 3-Methyl-butanal (2.2), (Z)-3-hexanol (2.9), (E,E)-2,4-Heptadienal (122.8), Benzeneacetaldehyde (1.9), 1-Methyl-4-(1-methylethylidene)-cyclohexene (1.5)	[4]
White tea (combined withering)	HS-SPME	GC-MS/OAV	2-heptanol (343.5), linalool (68.8), geraniol (21.8), dipentene (12.7), jasmone (10.1), methyl salicylate (4.0), (E)-β-damascenone (1.3), 1-nonanol (100.0)	[6]
Black tea (sun withering)	SPME/SAFE	GC-O/AEDA	Geraniol (785), (Z)-4-heptenal (644), 1-octen-3-ol (450), (E)-β-ionone (147), linalool (161), (E,E)-2,4-decadienal (59), (E,E)-2,4-nonadienal (30), (E)-2-nonenal (4)	[3]
Green tea (sun spreading)	HS-SPME	GC-MS/OAV	linalool (89.1–145.9), phenylethyl alcohol (436.7–561.1), phenylacetaldehyde (44.3–74.9), (E,Z)-2,6-nonadienal (887.0–903.8), (E,E)-2,4-nonadienal (49.2–57.5), (E,E)-2,4-decadienal (89.8–101.9), β-damascone (209.6–901.0), β-ionone (291.1–358.9)	[48]
Green tea (sun drying)	SPME	GC-MS/OAV	β-ionone (585.7), myrcene (1.7), linalool (7.2)	[34]
White tea (sun drying)	HS-SPME	OAV	2-heptanol (100.0), 1-octen-3-ol (5.2), linalool (12.7), geraniol (2.4), decanal (1.3), methyl salicylate (1.0)	[37]
White tea (shade-sun drying)	HS-SPME	OAV	2-heptanol (100.0), 1-octen-3-ol (3.5), linalool (11.7), geraniol (1.6), decanal (1.2)	[37]
Dark tea (sun drying)	HS-SPME	GC-MS/OAV	6-vinyldihydro-2,2,6-trimethylpyran-3(4H)-one (1051.3), trans-linalool oxide (furanoid) (870.4), cis-linalool oxide (furanoid) (293.3), trans-Linalool oxide (pyranoid) (196.3), nonanal (172.1), cis-linalool oxide (pyranoid) (66.8), D-limonene (4.3), eucalyptol (2.9)	[39]
Black tea (sun drying)	HS-SPME	GC-MS/GC-O	(E)-β-Damascenone (61,538.8), Linalool (354.1), β-ionone (41,851.8), dihydro-β-ionone (24,856.4), α-Ionone (168.7), Geraniol (163.5), Phenylethyl alcohol (74.0), (E)-2-Decenal (63.9), Methyl salicylate (49.2), Benzeneacetaldehyde (41.2), (E)-Nerolidol (20.7), Linalool oxide II (18.6), 1-Octen-3-ol (15.7), 1-Methyl-naphthalene (11.6), β-Cyclocitral (11.6), Hexanal (7.7),2-Pentyl-furan (7.1), Linalool oxide I (5.1), D-Limonene (3.5), α-Terpineol (2.5), Geranyl acetone (1.8)	[47]

Note: GC-O, gas chromatography olfactometry. AEDA, aroma extract dilution analysis. OAV, odor activity value.

## Data Availability

This study does not cover availability of data and material and code availability part.
